# Physicochemical and Antioxidant Alterations of Modified and Free Epigallocatechin Gallate Under Thermal Treatment in Air and Vacuum

**DOI:** 10.3390/jfb17010018

**Published:** 2025-12-26

**Authors:** Lianjing Yu, Zi Deng, Masato Nakagawa, Shanshan Zheng, Jun-ichiro Jo, Tomonari Tanaka, Yoshitomo Honda

**Affiliations:** 1 Department of Oral Anatomy, Osaka Dental University, 8-1 Kuzuhahanazono-cho, Hirakata 573-1121, Japan; yuyanjings@gmail.com (L.Y.); dengzi1014@gmail.com (Z.D.); m-nakagawa@cc.osaka-dent.ac.jp (M.N.); 33of1213@gmail.com (S.Z.); 2 Department of Biomaterials, Osaka Dental University, 8-1 Kuzuhahanazono-cho, Hirakata 573-1121, Japan; jo-j@cc.osaka-dent.ac.jp; 3 Department of Biobased Materials Science, Graduate School of Science and Technology, Kyoto Institute of Technology, Matsugasaki, Sakyo-ku, Kyoto 606-8585, Japan; t-tanaka@kit.ac.jp

**Keywords:** epigallocatechin gallate, antioxidant, thermal treatment, vacuum heating, DPPH assay, ABTS assay, gelatin, β-TCP

## Abstract

Epigallocatechin gallate (EGCG)—the most abundant catechin in green tea—is a promising component of advanced composite biomaterials. The pharmacological activity of EGCG is typically attenuated upon thermal processing, although the exact effects of heating free and modified EGCG in air and vacuum are unknown. To bridge this gap, we herein examined the effects of heating free and modified (in gelatin containing beta-tricalcium phosphate granules) EGCG in vacuum and air (100–220 °C, 1–16 h) on its physicochemical and antioxidant properties using water and ethanol solubility measurements, discoloration and antioxidant activity (2,2-diphenyl-1-picrylhydrazyl (DPPH) and 2,2′-azino-bis-3-ethylbenzothiazoline-6-sulfonic acid) assays, ultraviolet–visible spectroscopy, mass spectrometry, nuclear magnetic resonance spectroscopy, and attenuated total reflectance Fourier transform infrared spectroscopy. The antioxidant activity of EGCG-modified gelatin sponges was assessed in vitro using the DPPH assay and in vivo using a calvarial bone defect model in eight-week-old male Sprague–Dawley rats. Free and modified EGCG showed antioxidant activities, which were largely retained after heating in vacuum at 150 °C. These findings show that appropriate heating procedures preserve the antioxidant activity of EGCG and provide insights for the development of EGCG-based biomaterials.

## 1. Introduction

Tea prepared from the leaves of *Camellia sinensis* contains various polyphenols, such as catechins and their derivatives, which exhibit diverse pharmacological effects and health benefits. Hence, tea is widely consumed worldwide as a beverage and health food, especially in Asia [1,2], where green tea sales are expected to exceed USD 3.14 billion in 2024 [3]. Epigallocatechin gallate (EGCG, a catechin) is the most abundant [1] and pharmacologically active [4] component of green tea, exhibiting antioxidant [4,5], anti-inflammatory [6], antibacterial [7], and anticancer [8] activities, promoting osteoblastic differentiation [9] and growth factor release for dental diseases [10], and inhibiting osteoclastogenesis [11]. 

EGCG is a polyphenol containing four substituted rings (A–D). Rings B and D exhibit a higher physiological activity because of their multiple hydroxyl groups [12], which play an important role in protecting cells and tissues from oxidative stress [5]. However, these pharmacological effects are readily attenuated by various stimuli, including physicochemical (heating in water [13] and irradiation [14]) and chemical [15] factors. Although the pharmacological effects of EGCG have been extensively characterized, most studies have focused on its consumption in the form of tea [16]. The scope of EGCG applications in medicine and dentistry has been broadened through its hybridization with various materials, including polymers (gelatin, collagen, hyaluronic acid, chitosan, and polycaprolactone) [17,18,19,20,21,22], metals (gold nanoparticles) [23,24], and inorganic nanomaterials (hydroxyapatite, Fe_2_O_3_, and TiO_2_) [25,26,27]. Furthermore, extensive efforts in nanoparticle research have expanded EGCG applications as a functional drug in drug delivery systems [28,29,30]. In addition to the effects of EGCG mentioned above, these composites have a broad application scope and can exhibit anticancer [23,24,28] and antibacterial [21] properties, regulate senescence-associated secretory phenotype factors from senescent cells [31], act as stem cell seeding carriers [31], and promote bone regeneration [17,19,22].

Our group has developed EGCG-conjugated materials for bone regeneration, including EGCG-conjugated gelatin with or without β-tricalcium phosphate (β-TCP) [20,31,32]. Gelatin was subjected to dehydrothermal treatment (vacuum heating) in the presence of EGCG to enhance crosslinking and obtain an optimal scaffold for cells. The resulting materials showed a very robust crosslinked network and exhibited a remarkably augmented bone-forming ability [20,31] and antisenescence activity even after heating [31]. Although studies dealing with the vacuum heating of EGCG-containing biomaterials are few, hydrothermal treatment has been applied to EGCG-capped α-Fe_2_O_3_ nanoparticles [26] and EGCG/Zn-coated Ti [33].

The thermal and digestion treatments of free EGCG cause its degradation through epimerization and autoxidation and afford diverse products, such as monomeric catechins, quinone derivatives, and dimerized oxidation products (theasinensin A, D, and P-2) [34,35]. Specifically, when heated in water, EGCG undergoes accelerated decomposition [12,34,36,37] and loses its antioxidant capacity [13]. For example, 50% of EGCG in aqueous solution undergoes notable decomposition after 40 min of heating at 120 °C [37]. After 45 min of heating at 180 °C in water (using oil bath), total phenolic content decreases by >50%, and the rate of 2,2-diphenyl-1-picrylhydrazyl (DPPH) radical scavenging decreases by 90% [13]. The stability of catechins in solution generally depends on pH and temperature [37]. Oxygen concentration, which relates to oxidation, is an important factor promoting instability [36], and the oxidation of EGCG is closely attributed to peroxyl radicals generated from atmospheric oxygen [38].

The application of heating for EGCG and EGCG-based biomaterials in air or under vacuum possibly increase, as with water-based heating. However, comprehensive insights into the changes in the physicochemical properties and antioxidant ability of EGCG upon heating in a vacuum are currently scarce. The aim of this study is to evaluate the effects of vacuum and atmospheric heating at different durations and temperatures on the physicochemical and antioxidant properties of free EGCG. Furthermore, to determine whether modified EGCG on biomaterials exhibits similar effects, the antioxidant properties of EGCG-conjugated gelatin sponges containing β-TCP were evaluated both in vivo and in vitro after vacuum heating as a model experiment.

## 2. Materials and Methods

### 2.1. VHT and AHT

EGCG powder (Taiyo Kagaku Co., Ltd., Tokyo, Japan) was provided by Toagosei Co., Ltd. (Tokyo, Japan). Samples with non-heating treatment were denoted as NT. For each thermal treatment, the EGCG powder was placed into four separate amber glass vials, which were heat-treated independently (*n* = 4). Vacuum heating treatment (VHT) was performed using a vacuum dryer (AVO-250NS, AS ONE Co., Osaka, Japan), and atmospheric heating treatment (AHT) was performed using a convection oven (LC-122, TABAI ESPEC Co., Osaka, Japan). To investigate the effect of temperature, samples were heated for 4 h at 100–220 °C (VHT) and 100–200 °C (AHT). To evaluate the effect of treatment duration, samples were subjected to VHT or AHT at 150 °C for 1–16 h. Hereafter, vacuum and atmospheric heating treatment are designated as VHT or AHT (temperature, time), e.g., vacuum heating treatment at 150 °C for 16 h: VHT (150 °C, 16 h). All samples were sealed and stored at −20 °C after heating and dissolved as needed for assays. A stereomicroscope (SZ61; Olympus Corporation, Tokyo, Japan) and digital camera (GR III, RICOH Company, Ltd., Tokyo, Japan) were used for macro photography. Color differences (ΔE, CIEDE2000) were measured using a spectrophotometer (Spectro 1 Pro, Variable, Chattanooga, TN, USA) and black art paper as a reference. Relative ΔE (%) was calculated as 100% × (ΔE of experimental group/ΔE of EGCG with NT).

### 2.2. Antioxidant Activity Assay

Antioxidant activity was measured using 2,2′-azino-bis-3-ethylbenzothiazoline-6-sulfonic acid (ABTS, a methemoglobin oxidase inhibitor) and 2,2-diphenyl-1-picrylhydrazyl (DPPH). The ABTS assay was performed following manufacturer’s instructions (709001; Cayman Chem. Co., Ann Arbor, MI, USA): EGCG powder was dissolved in antioxidant assay buffer (1×) to a concentration of 50 μg/mL. Absorbance (Abs) at 405 nm was measured using a microplate reader (SpectraMax M5, Molecular Devices, San Jose, CA, USA). ΔAbs values were calculated as the difference in absorbance between each sample and the blank. The rate of antioxidant activity retention was calculated as 100% × (ΔAbs of experimental group/ΔAbs of EGCG with NT).

The DPPH assay was performed using the corresponding kit (D678, Dojindo Lab., Kumamoto, Japan) according to manufacturer’s protocol. EGCG powder was dissolved in 80% aqueous ethanol (EtOH) to a concentration of 5 μg/mL, and the solution was mixed with the DPPH working solution in a 96-well plate and incubated at 25 °C in the dark. After 30 min, absorbance at 517 nm was measured using a microplate spectrophotometer (SpectraMax M5). The rate of antioxidant activity retention was calculated as 100% × (ΔAbs of experimental group/ΔAbs of EGCG with NT).

For sponges (see Section 2.4), 80% EtOH was premixed with the DPPH working solution. One-quarter of the sponge (~9 mg) was added to 400 μL of the premixed solution and ground at 30 times/s for 2 min with 0.8 mm–diameter glass beads (Toshinriko, Tokyo, Japan) using a mixer mill (MM301, Retsch, Haan, Germany) (*n* = 4). The mixture was centrifuged, the supernatant transferred to a 96-well plate, and absorbance at 517 nm measured in the dark using a microplate spectrophotometer (SpectraMax M5).

### 2.3. Physicochemical Characterization

Ultraviolet–visible (UV–vis) spectra were obtained using a Nanodrop spectrophotometer (ND1000, Thermo Fisher Scientific, Waltham, MA, USA). Mass spectrometry analysis (JMS-700k, JEOL Ltd., Tokyo, Japan) was conducted using m-nitrobenzyl alcohol as the matrix. ^1^H nuclear magnetic resonance (NMR) spectra (BioSpin AV-600, Bruker Co., Billerica, MA, USA) were recorded in deuterium oxide. Attenuated total reflection Fourier transform infrared (ATR-FTIR) spectra (FT/IR-4600, JASCO Co., Tokyo, Japan) were recorded in the wavenumber range of 400–4000 cm^−1^. Powder X-ray diffraction (XRD) patterns (XRD-6000, Shimadzu Co., Kyoto, Japan) were collected within the range of 2*θ* = 20–40°.

### 2.4. Preparation of Gelatin Sponges

Vacuum-heated gelatin sponges containing 80 wt% porous β-tricalcium phosphate (β-TCP) particles (500–1000 μm) (vh-GSβ), vacuum-heated chemically synthesized gelatin sponges containing 80 wt% porous β-TCP particles (vhc-GSβ), and vacuum-heated chemically synthesized gelatin sponges containing 0.28 wt% intact EGCG and 80 wt% porous β-TCP particles (vhEc-GSβ) were prepared as described elsewhere [32] and provided by Toagosei Co., Ltd. The specimen size was fixed to 9 mm × 1 mm, and VHT was conducted at 150 °C for 16 h.

### 2.5. Sponge Morphology Observation

A stereomicroscope (SZ61; Olympus Corporation) was used for macro photography. Surface microstructure was observed using field-emission scanning electron microscopy (FE-SEM; S-4800; Hitachi, Ltd., Tokyo, Japan) at an acceleration voltage of 5 kV. Prior to FE-SEM imaging, all samples were coated with O_s_O_4_ using an osmium coating device (HPC-20 Device; Vacuum Device Co., Ltd., Ibaraki, Japan). Pore size was quantified as the maximum Feret diameter extracted from binary FE-SEM images using the ImageJ software (v. 1.54g, National Institutes of Health, Bethesda, MD, USA). Pores with a minimum Feret diameter of <10 μm were excluded because of their insufficiency for cellular infiltration [39].

### 2.6. Animal Experiments

Animal experiments were conducted with the approval of the Osaka Dental University Ethics Committee (Approval No. 25-06005). Sixteen eight-week-old male Sprague–Dawley rats purchased from Shimizu Laboratory Supplies Co., Ltd. (Kyoto, Japan) were used to construct a 9 mm-diameter critical-sized bone defect at calvaria under deep anesthesia. All animals were housed in a well-maintained animal facility under standard experimental conditions throughout the study period. No specific inclusion or exclusion criteria were established, and all animals used in the experiment were included in the analysis. The animals were randomly divided into four groups: control, vh-GSβ, vhc-GSβ, and vhEc-GSβ (*n* = 4 per group), with the experimental unit defined as a single animal. In the experimental groups, a single hydrogel sheet (vh-GSβ, vhc-GSβ, or vhEc-GSβ) prepared using Ringer’s solution (90 μL) was implanted into the defect site, while the control group received no material. Humane endpoints were defined as feeding difficulty, abnormal posture, or >20% weight loss; however, no animals reached these endpoints. All rats underwent cardiac perfusion fixation with a 4% polyformaldehyde solution (No. 15291; Muto Pure Chemicals Co., Ltd., Tokyo, Japan) on the third postoperative day. Samples were immersed in Morse solution (135-17071; FUJIFILM Wako Pure Chemical Corporation, Osaka, Japan) for three days for decalcification and then subjected to frozen-tissue sectioning using the Kawamoto method [40]. Sections were cut at a thickness of 8 μm (Leica CM1950, Leica Biosystems, Wetzlar, Germany).

### 2.7. Immunofluorescence Staining

Frozen sections of animal tissues were blocked and permeabilized in phosphate-buffered saline (164-28713; FUJIFILM Wako Pure Chemical Corporation, Osaka, Japan) containing 5% goat serum (S-1000, Vector Laboratories, Inc., Newark, CA, USA) and 0.3% Triton X-100 (Code 12967-32, Nacalai, Kyoto, Japan) for 30 min. Immunostaining was performed using an anti-4-hydroxynonenal (4-HNE) polyclonal antibody (bs-6313R-Cy5, Bioss Antibodies, Woburn, MA, USA; 1:100) in the dark at 4 °C overnight. After washing three times with phosphate-buffered saline, the cell nuclei were stained with 4′,6-diamidino-2-phenylindole (DAPI) Fluoromount-G (Southern Biotech, Birmingham, AL, USA). Images were captured using a digital microscope (BZ-800; Keyence, Osaka, Japan). Each experimental group contained four animals, and three random sections per animal were analyzed for statistical evaluation. The fraction of 4-HNE-positive staining was determined using immunofluorescence images acquired at a magnification of 10×. Regions of interest (ROIs) were defined within the calvarial defect area in each section. For groups with material implantation, ROIs were restricted to material-filled defect regions. Quantitative analysis was performed based on these predefined ROIs using the ImageJ software (v. 1.54g), with the quantitative metric calculated as area of 4-HNE fluorescence/area of DAPI fluorescence (*n* = 4).

### 2.8. Statistical Analysis

Data processing and analysis were performed using the Prism 10.2.1 software (GraphPad Software, San Diego, CA, USA). Data normality was assessed using the Shapiro–Wilk test, and the homogeneity of variances was evaluated using the Brown–Forsythe test. For comparisons involving more than two groups, one-way analysis of variance (ANOVA) followed by Tukey’s multiple comparisons test was used when data met the assumptions of normality and homogeneity of variances. When normality was satisfied, but variances were unequal, Welch’s ANOVA followed by Dunnett’s T3 multiple comparisons test was applied. When data were not normally distributed, the Kruskal–Wallis test followed by Dunn’s multiple comparisons test was used. For comparisons between two groups, Welch’s *t* test was applied when normality was satisfied but variances were unequal. All results were presented as means ± standard deviations. Differences were considered statistically significant at *p* < 0.05.

### 2.9. Use of Artificial Intelligence

During the preparation of this manuscript, the authors used Paperpal (v. 4.24.0, Cactus Communications Pvt, Ltd., Mumbai, Maharashtra, India) for English language polishing. After using this tool, the authors reviewed and edited the content as required and take full responsibility for the writing of this article and its publication.

## 3. Results

### 3.1. Color Change of Free EGCG After Heat Treatment

**Figure 1:**
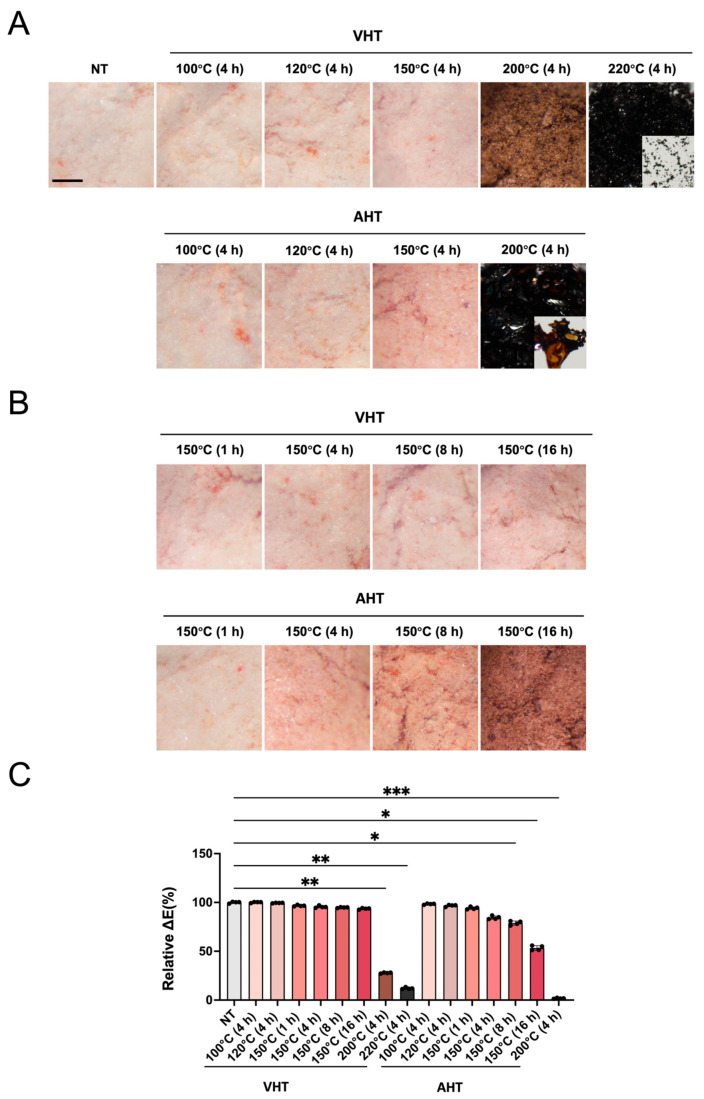
Stereomicroscopic and quantitative analysis of epigallocatechin gallate (EGCG) powder treated with or without heating at different temperatures and durations. (**A**) Stereomicroscopic images of EGCG treated at different heating temperatures for 4 h. NT: non-heating treatment; VHT: vacuum heating treatment; AHT: atmospheric heating treatment. Insets: the dispersions of corresponding samples. Scale bar = 500 μm. (**B**) Stereomicroscopic images of EGCG heated at 150 h for different durations. (**C**) Quantitative analysis of color differences. *n* = 4; Welch’s ANOVA with Dunnett’s T3 multiple comparisons test for comparisons with the control group; * *p* < 0.05, ** *p* < 0.01, *** *p* < 0.001.

EGCG without heating (NT) exhibited a pale pinkish-white color (Figure 1A). The increase in temperature augmented the discoloration for both EGCG with VHT and AHT. EGCG with VHT (200 °C, 4 h) appeared brown, while EGCG with AHT (200 °C, 4 h) was black and lost its powdery appearance, exhibiting a plate-like particle morphology (Figure 1A). EGCG with VHT (220 °C, 4 h) was nearly black yet maintained a powdery state (Figure 1A). At 150 °C, the pink color of EGCG with VHT minimally intensified with an increase in heating time from 1 to 16 h (Figure 1B, upper part). AHT caused a more pronounced color change than VHT when heating was performed for ≥4 h; in particular, EGCG with AHT (150 °C, 16 h) was brown and completely different in color from EGCG with VHT (150 °C, 16 h) (Figure 1B). These observations were supported by the quantitative color differences (Figure 1C).

### 3.2. Solubility Behavior of Free EGCG in Ultrapure Water (UPW) and EtOH

**Figure 2:**
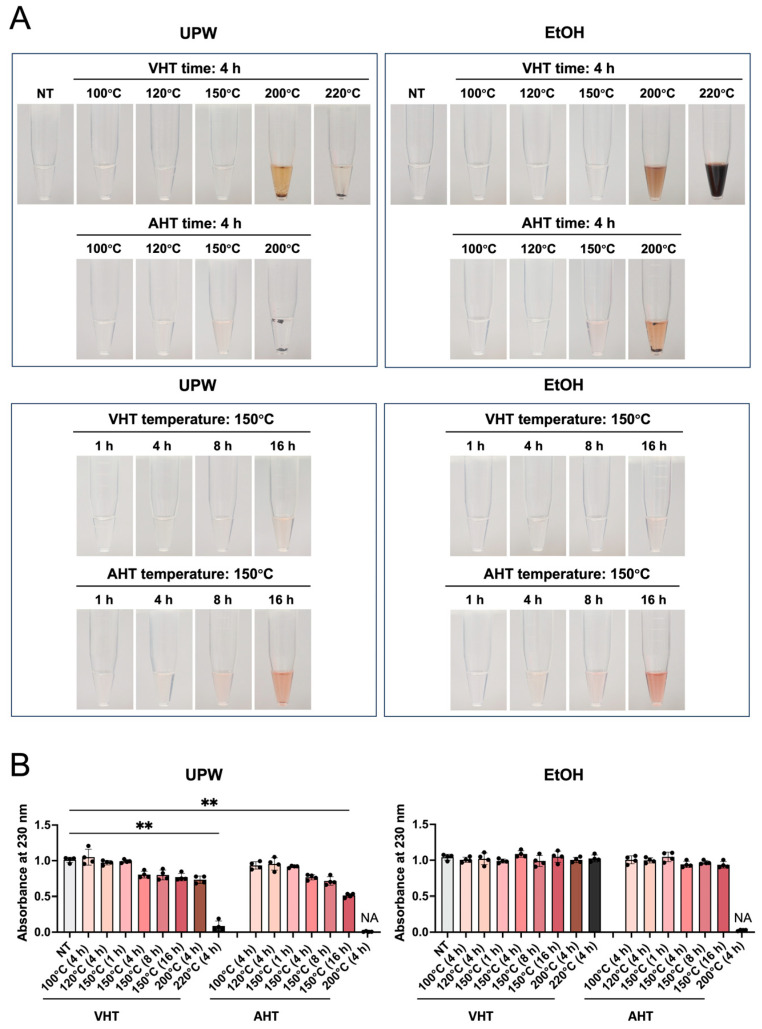
Solubility of EGCG powder treated with or without heating at different temperatures and durations. UPW: ultrapure water; EtOH: 80% ethanol. NT: non-heating treatment; VHT: vacuum heating treatment; AHT: atmospheric heating treatment. (**A**) Representative images showing the solubility of EGCG after different treatments. (**B**) Quantitative analysis of Ultraviolet-visible (UV–vis) spectra absorbance at 230 nm for EGCG-dissolved solution. NA = not available. **Left** (UPW): Kruskal–Wallis test with Dunn’s multiple comparisons test; **Right** (EtOH): Welch’s ANOVA with Dunnett’s T3 multiple comparisons test. *n* = 4; ** *p* < 0.01. Statistical analyses were calculated for comparisons with the control group.

The solubility of EGCG in the solvent appears to affect the process of combining this molecule with biomaterials. Figure 2 shows images of the aqueous and ethanolic solutions of heat-treated EGCG. EGCG with NT and EGCG subjected to VHT at 100–120 °C for 4 h and 150 °C for 1 h completely dissolved in UPW and EtOH to form transparent solutions (Figure 2A). This conclusion was supported by UV–vis spectroscopic data (Figure 2B). EGCG subjected to VHT above 200 °C partially precipitated in UPW but fully dissolved in EtOH to yield a brown solution (Figure 2A), although quantitative analysis did not show significant differences (Figure 2B). EGCG with VHT (220 °C, 4 h) was poorly soluble in UPW, causing its slight discoloration, yet dissolved in EtOH to yield a black solution (Figure 2A). EGCG with AHT (150 °C, 4 h and 8 h) dissolved in UPW and EtOH to afford light pink solutions, whereas a notably deeper color was obtained when heating was performed for 16 h (Figure 2A). EGCG with AHT (200 °C, 4 h) was insoluble in UPW and almost insoluble in EtOH, affording an orange liquid (Figure 2A,B).

### 3.3. Antioxidant Activity of Free EGCG After Heat Treatment

**Figure 3:**
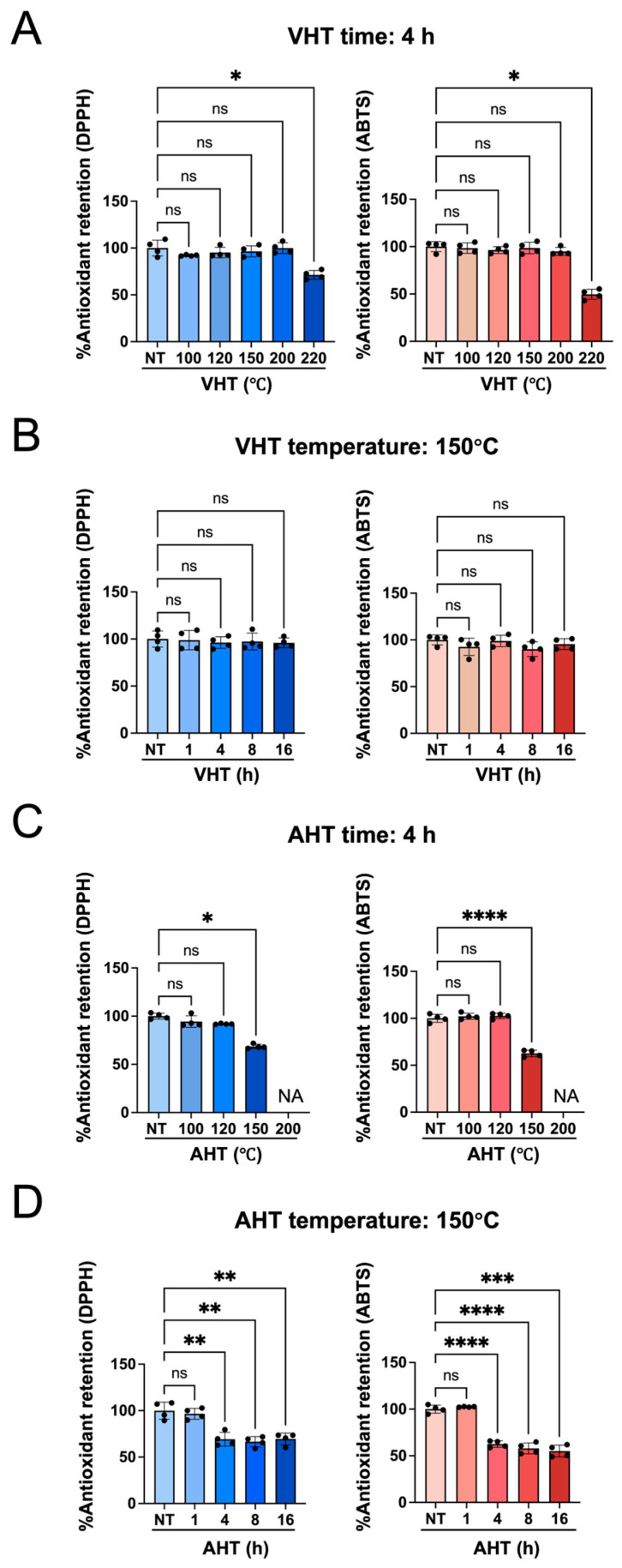
Antioxidant activity of EGCG subjected to different treatments. NT: non-heating treatment; VHT: vacuum heating treatment; AHT: atmospheric heating treatment. (**A**–**D**) **Left**: 2,2-diphenyl-1-picrylhydrazyl (DPPH) assay; **right**: 2,2′-azino-bis-3-ethylbenzothiazoline-6-sulfonic acid (ABTS) assay. NA = not available. *n* = 4; ns: not significant; * *p* < 0.05; ** *p* < 0.01; *** *p* < 0.001; **** *p* < 0.0001. (**A**) EGCG treated with VHT for 4 h at different temperatures; Kruskal–Wallis test with Dunn’s multiple comparisons test. (**B**) EGCG treated with VHT at 150 °C for different heating durations; Welch’s ANOVA with Dunnett’s T3 multiple comparisons test (**left**) and Kruskal–Wallis test with Dunn’s multiple comparisons test (**right**). (**C**) EGCG treated with AHT for 4 h at different temperatures; Kruskal–Wallis test with Dunn’s multiple comparisons test (**left**) and Welch’s ANOVA followed by Dunnett’s T3 multiple comparisons test (**right**). (**D**) EGCG treated with AHT at 150 °C for different heating durations; Welch’s ANOVA followed by Dunnett’s T3 multiple comparisons test. All statistical analyses were calculated for comparisons with the control group.

The effects of heating on the antioxidant activity of EGCG are presented in Figure 3. Four-hour VHT had negligible effects when performed at ≤200 °C but significantly decreased antioxidant activity when performed at 220 °C (Figure 3A). EGCG retained its antioxidant activity after up to 16 h of VHT at 150 °C (Figure 3B), whereas AHT at 150 °C significantly reduced the antioxidant activity of EGCG after only 4 h (Figure 3C,D).

### 3.4. Physicochemical Properties of Free EGCG After Heat Treatment

**Figure 4:**
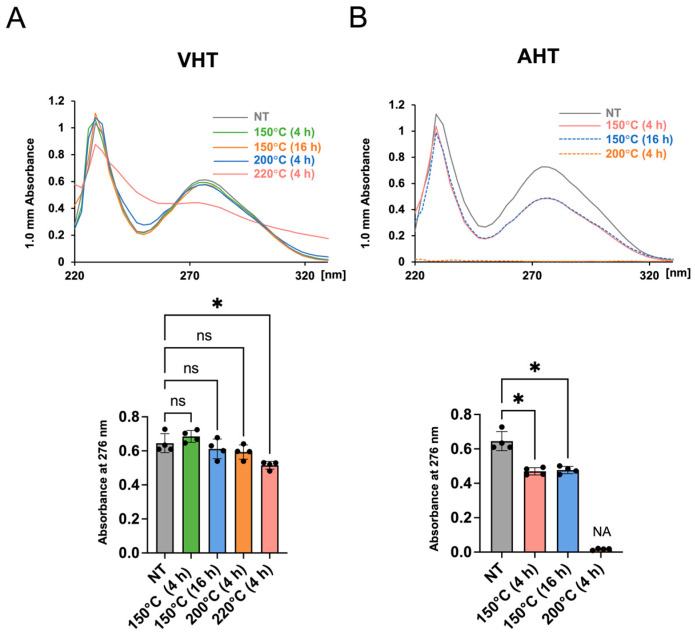
The UV–vis analysis and its quantitative data of EGCG treated with or without heating at different temperatures and durations. NT: non-heating treatment; VHT: vacuum heating treatment; AHT: atmospheric heating treatment. (**A**) Absorbance of EGCG after different treatments under VHT (**top**) and quantification of absorbance at 276 nm (**bottom**). (**B**) Absorbance of EGCG after different treatments under AHT (**top**) and quantification of absorbance at 276 nm (**bottom**). NA = not available. *n* = 4; Welch’s ANOVA with Dunnett’s T3 multiple comparisons test for comparisons with the control group; ns: not significant, * *p* < 0.05.

To explore the mechanisms underlying the heating-induced decrease in antioxidant activity, we evaluated the UV–vis spectra of heated EGCG and quantified absorbance at 276 nm. The absorption spectra of VHT samples almost matched those of EGCG without treatment in all cases except EGCG with VHT (220 °C, 4 h); at that high temperature, absorbance at 276 nm was markedly decreased (Figure 4A). In the case of AHT, absorbance at 276 nm significantly decreased upon heating at 150 °C for 4 h and 16 h. We could not obtain data for EGCG with AHT (200 °C, 4 h), possibly because of its low solubility (Figure 4B).

**Figure 5:**
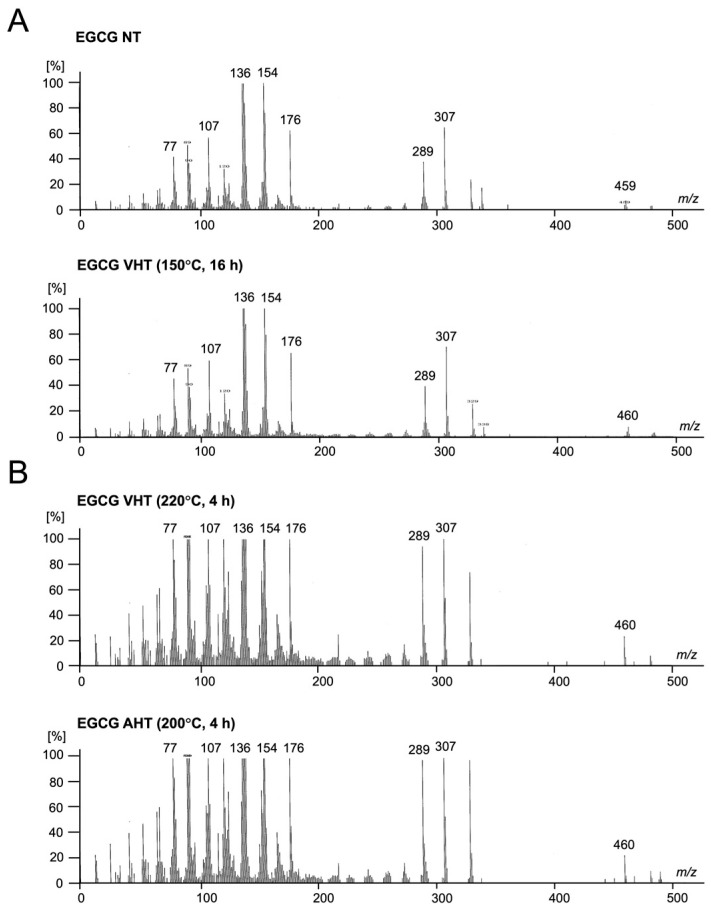
Mass spectrometric analysis of EGCG treated with or without heating at different temperatures and times. NT: non-heating treatment; VHT: vacuum heating treatment; AHT: atmospheric heating treatment. (**A**) **Top**: EGCG with NT; **bottom**: EGCG with VHT (150 °C, 16 h). (**B**) **Top**: EGCG with VHT (220 °C, 4 h); **bottom**: EGCG with AHT (200 °C, 4 h).

**Figure 6:**
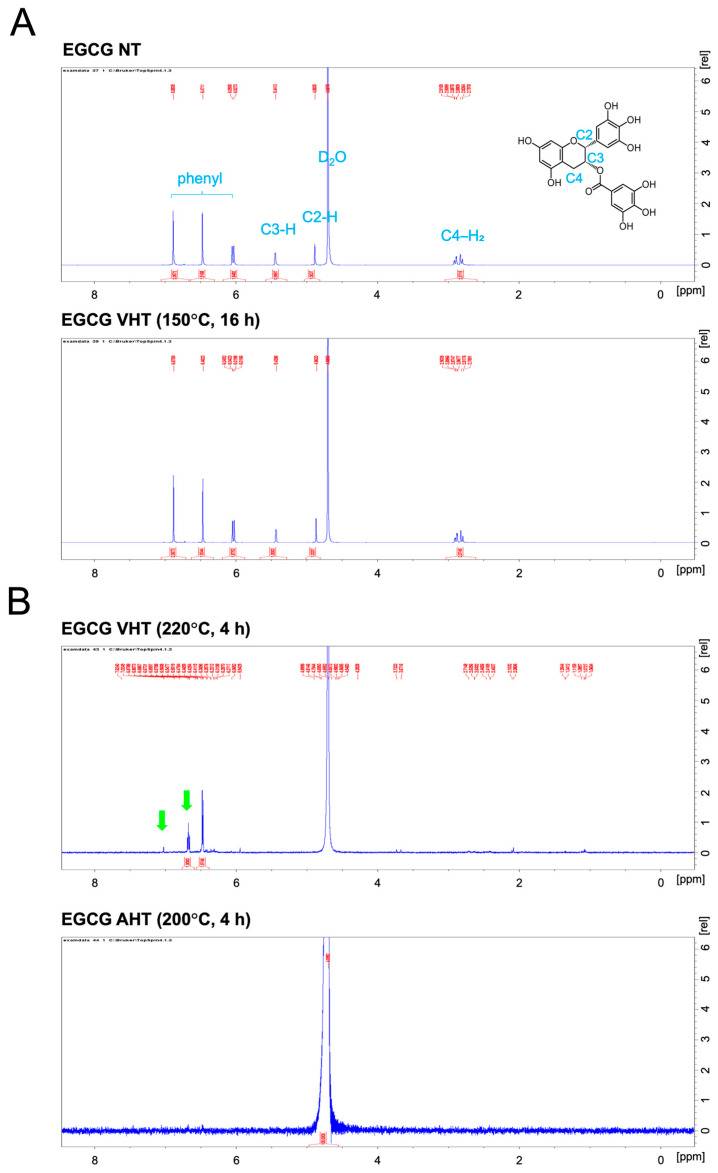
^1^H Nuclear Magnetic Resonance analysis of EGCG treated with or without heating at different temperatures and times. NT: non-heating treatment; VHT: vacuum heating treatment; AHT: atmospheric heating treatment. (**A**) **Top**: EGCG with NT; **bottom**: EGCG with VHT (150 °C, 16 h). (**B**) **Top**: EGCG with VHT (220 °C, 4 h), green arrows: peaks at 6.7 and 7.0 ppm; **bottom**: EGCG with AHT (200 °C, 4 h), not detectable due to poor solubility.

**Figure 7:**
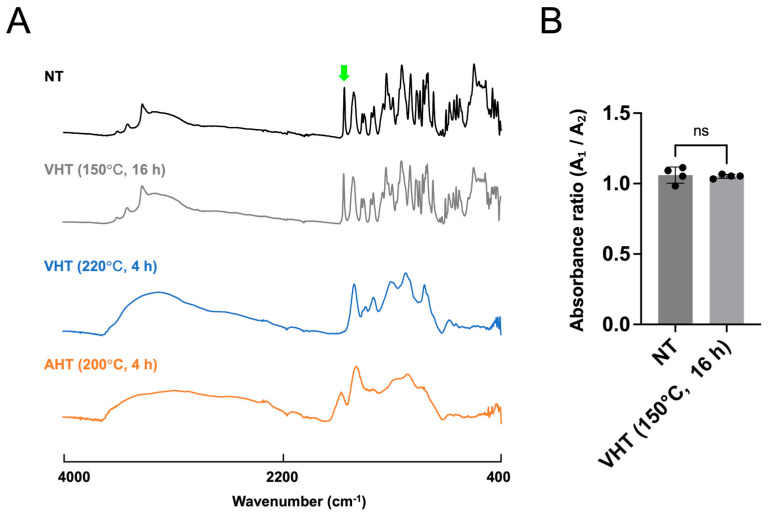
Attenuated total reflection-Fourier transform infrared (ATR–FTIR) spectroscopic analysis of EGCG powder treated with or without heating at different temperatures and times. NT: non-heating treatment; VHT: vacuum heating treatment; AHT: atmospheric heating treatment. (**A**) ATR–FTIR spectra of EGCG powder under the indicated treatment conditions. From top to bottom: EGCG with NT; EGCG with VHT (150 °C, 16 h); EGCG with VHT (220 °C, 4 h); EGCG with AHT (200 °C, 4 h). Green arrow: peak at 1689 cm^−1^ tentatively assigned to C=O bond. (**B**) Ratio of absorbance at 1689 cm^−1^ (A_1_, peak of interest) to that at 1613 cm^−1^ (A_2_, aromatic C=C stretching vibration used as an internal reference). *n* = 4; Welch’s *t* test; ns: not significant.

Subsequently, we performed mass spectrometric, NMR spectroscopic, and ATR-FTIR spectroscopic analyses to probe the physicochemical properties of EGCG treated at 150 °C for 16 h with vacuum heating as well as to determine instability of EGCG with VHT (220 °C, 4 h) and AHT (200 °C, 4 h) (Figure 5, Figure 6 and Figure 7). This is because the former sample showed high stability, whereas the latter differed remarkably from intact EGCG. Mass spectrometry, NMR spectroscopy, and ATR-FTIR spectroscopy revealed no notable differences between EGCG with NT and with VHT (150 °C, 16 h) (Figure 5A, Figure 6A and Figure 7). However, EGCG with VHT (220 °C, 4 h) and AHT (200 °C, 4 h) exhibited distinct decomposition patterns, producing more low-*m*/*z* fragments than EGCG with NT and with VHT (150 °C, 16 h) (Figure 5B). The ^1^H NMR spectra of EGCG with NT and with VHT (150 °C, 16 h) showed signals at 2.7–3.0 ppm (C-ring CH_2_), 4.9 and 5.5 ppm (C-ring CH), 6.0 ppm (A-ring CH), 6.5 ppm (B-ring CH), 6.9 ppm (D-ring CH) in agreement with a previous study [41]. Unlike the spectrum of EGCG with VHT (150 °C, 16 h), that of EGCG with VHT (220 °C, 4 h) had very weak A-ring signals, and no C-ring and D-ring signals were observed, whereas the B-ring signals were detectable. Two new signals appeared at 6.7 and 7.0 ppm (Figure 6B, green arrows). The specific peak of EGCG, as mentioned above, was undetectable in the spectrum of EGCG with AHT (200 °C, 4 h), possibly because this sample was insoluble in D_2_O (Figure 6B). Compared with those of EGCG with NT and with VHT (150 °C, 16 h), the ATR-FTIR spectra of EGCG with VHT (220 °C, 4 h) and with AHT (200 °C, 4 h) featured broader peaks in the range of 400–1700 cm^−1^, where the peaks of EGCG with NT were located [29]. The peak at 1689 cm^−1^ tentatively assigned to C=O bonds (Figure 7A, green arrow) remarkably weakened upon VHT at 220 °C for 4 h. The ratio of absorbance at 1689 cm^−1^ to that at 1613 cm^−1^ was not significantly different between EGCG with NT and EGCG with VHT (150 °C, 16 h) (Figure 7B).

### 3.5. In Vivo and In Vitro Antioxidant Effects of EGCG-Modified Gelatin

**Figure 8:**
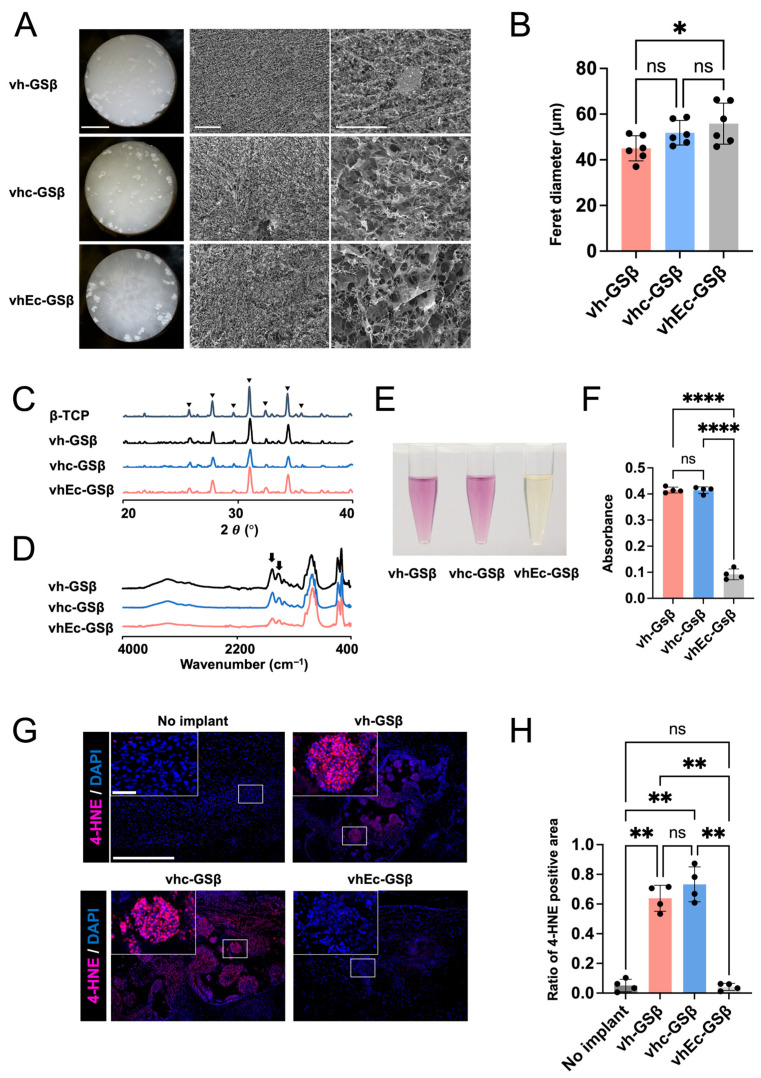
Characterization and antioxidant activity of samples. (**A**) Three types of sponges: vacuum-heated (150 °C, 16 h) gelatin sponges containing β-tricalcium phosphate (β-TCP) granules (vh-GSβ), vacuum-heated chemically synthesized gelatin sponges containing β-TCP (vhc-GSβ), and vacuum-heated chemically synthesized gelatin sponges containing EGCG and β-TCP (vhEc-GSβ). Macroscopic appearances (**left**; scale bar = 5 mm) and field-emission scanning electron microscopic appearances (**middle**; scale bar = 500 µm, **right**; scale bar = 200 µm). (**B**) Quantitative analysis of pore size based on the average of the maximum Feret diameter. *n* = 6; one-way ANOVA with Tukey’s multiple comparisons test; ns: not significant; * *p* < 0.05. (**C**) X-ray powder diffraction patterns. Arrow heads: the characteristic diffraction peaks of β-TCP. (**D**) ATR-FTIR spectra of the samples. Arrows: the characteristic peaks of amide bands. (**E**) DPPH assay. (**F**) Quantitative analysis of DPPH scavenging activity using Welch’s ANOVA followed by Dunnett’s T3 multiple comparisons test. *n* = 4, ns: not significant, **** *p* < 0.0001. (**G**) Immunofluorescence staining for 4-Hydroxynonenal (4-HNE) in rat calvarial bone defects implanted with or without samples. Scale bars: **top** = 50 µm; **bottom** = 500 µm. (**H**) Quantitative analysis of the ratio of 4-HNE to 4′,6-diamidino-2-phenylindole (DAPI) fluorescence area. *n* = 4; Welch’s ANOVA followed by Dunnett’s T3 multiple comparisons test; ns: not significant; ** *p* < 0.01.

Depending on the modified state, the physicochemical properties of EGCG can change upon heat treatment. To confirm whether polymer (gelatin)-modified EGCG retains its antioxidant properties after VHT at 150 °C for 16 h, we prepared gelatin-based materials with (vhEc-GSβ) and without (vhc-GSβ and vh-GSβ) EGCG and compared their antioxidant properties in vitro and in vivo. All three types of sponges showed porous structures (Figure 8A) and contained β-TCP (Figure 8C) [42] and gelatin (Figure 8D) [43]. Quantitative analysis of sponge pore size based on FE-SEM images indicated only minor variations among the samples (Figure 8B).

In the DPPH radical scavenging assay, the reaction solutions of vh-GSβ and vhc-GSβ were deep purple, whereas that of vhEc-GSβ showed a significantly lighter pale-yellow color (Figure 8E). In agreement with these color changes, the DPPH absorbance (517 nm) was significantly lower for vhEc-GSβ than for vh-GSβ and vhc-GSβ, which indicates that EGCG retained its high DPPH radical scavenging activity upon modification with gelatin (Figure 8F).

β-TCP, a well-known bone substitute, promotes the generation of oxidative stress in vivo and in vitro [44,45,46]. To evaluate the in vivo antioxidant properties of the three types of sponges, we implanted them in a critical bone defect model at the rat calvaria (9 mm) and subjected them to immunofluorescence staining for a reactive oxygen species (ROS) marker (4-hydroxynonenal) at three days post-surgery. Strong staining was observed for the sponges without EGCG (vh-GSβ and vhc-GSβ), possibly at the inner part of β-TCP. vhEc-GSβ showed negligible staining despite having the same amount of β-TCP, which demonstrates that modified EGCG subjected to VHT at 150 °C for 16 h suppressed β-TCP-induced oxidative stress in vivo and retained its antioxidant properties (Figure 8G,H).

## 4. Discussion

Given the worldwide consumption of tea as beverages and foods, various studies have evaluated changes in EGCG properties upon heating, especially in aqueous media [13]. Recent advances in regenerative therapies have widened the application scope of EGCG to biomaterial use [17,18,19,20,21,22,23,24,25,26,27]. However, the changes in the properties of modified and free EGCG upon thermal treatment in vacuum remain underexplored. Herein, we evaluated the changes in the physicochemical properties and antioxidant activity of free EGCG upon VHT and AHT. Additionally, the antioxidant ability of modified EGCG in EGCG-conjugated gelatin containing β-TCP was evaluated in vivo and in vitro after vacuum heating. VHT caused negligible changes in the physicochemical and antioxidant properties of free EGCG when performed at 200 °C for 4 h and 150 °C for 16 h. EGCG modified with gelatin also retained its antioxidant capacity even after 16 h of VHT at 150 °C.

Color is an intuitive indicator of EGCG stability [34]. Upon VHT, the color of EGCG changed more gradually than upon AHT (Figure 1). Previous studies have reported similar color changes to pinkish-brown for EGCG powder stored for long periods under atmospheric conditions [34]. This color change may stem from colored compounds formed by oxidation, such as catechin diquinone or other catechin derivatives [34]. The enzyme-catalyzed oxidation of EGCG is known to yield colored products such as dehydrotheasinene and its quinoxaline derivatives [47]. Samples stored under low-humidity and high-oxygen conditions have been reported to exhibit increased theasinensin formation and color development compared with those stored under high-humidity conditions [34]. Although we could not isolate the components responsible for color changes after heating, AHT may cause the coloration of EGCG by promoting oxidation affording colored compounds. Discoloration change was observed even upon VHT, becoming more pronounced with increasing temperature and time. The low-humidity, low-oxygen conditions of VHT may promote the dehydrative condensation of EGCG and, hence, the formation of polymeric species. 

Previous studies have shown that the solubility of EGCG and other catechins partially depends on solvent polarity [48]. Herein, EGCG treated at higher temperatures exhibited reduced solubility. Moreover, the solubility of EGCG in EtOH exceeded that in UPW (Figure 2). Mass spectrometric, NMR spectroscopic, and ATR-FTIR spectroscopic analyses revealed differences in EGCG subjected to VHT (220 °C, 4 h) and AHT (200 °C, 4 h) (Figure 6 and Figure 7). EGCG subjected to VHT or AHT (particularly VHT above 220 °C or AHT above 200 °C) may undergo structural changes, such as oxidation and decomposition, which increase the amount of low-polarity small molecules. Those changes might elicit the alteration of solubility.

The antioxidant activity of free EGCG showed little change upon VHT (even at 200 °C for 4 h and 150 °C for 16 h) but remarkably decreased after AHT at 150 °C for 4 h (Figure 3C). The absorption peak of EGCG in the 270–276 nm region (*λ*_max_ ≈ 275 nm) is mainly due to the formation of a conjugated π electron system from multiple phenolic rings (especially the polyphenol and gallate group in ring D) [49,50]. With the decomposition of EGCG, this peak loses intensity [51]. Furthermore, epigallocatechin (EGC), which does not have a gallate group (D-ring) and has a lower antioxidant activity than EGCG, features a notably weaker absorbance at ~270 nm at the same concentration [38,52,53]. Herein, the absorbance at 276 nm was significantly attenuated upon VHT (220 °C, 4 h) and AHT (150° C, 4 h and 16 h) (Figure 4). ATR-FTIR spectroscopic analysis showed that the C=O peak (1689 cm^−1^) lost intensity after VHT (220 °C, 4 h), possibly because of the degradation of the ester bond between the C- and D-rings and galloyl group detachment (Figure 7). Taken together, these results suggested that high-temperature VHT and AHT induced changes in the galloyl group.

The minimization of oxygen exposure through VHT resulted in antioxidant activity retention significantly exceeding that observed upon AHT (Figure 3). The effects of heating EGCG in water or under humid conditions on its antioxidant properties have been actively studied because of the widespread consumption of tea, the general consensus being that EGCG is heat-sensitive and loses its antioxidant activity upon heating [13]. Murakami et al. reported that the radical scavenging activity of EGCG almost vanished after 120 min of heating at 180 °C (oil bath) under aqueous conditions. The degradation of EGCG produces gallic acid and gallocatechin gallate (GCG, epimer of EGCG) [13]; during this degradation process, GCG is thought to be produced by the epimerization of EGCG, while gallic acid and EGC are produced by degalloylation [34]. Unlike that of EGC, the antioxidant activity of GCG is not remarkably lower than that of EGCG [54]. In our study, although EGCG decomposed at temperatures exceeding 220 °C, its antioxidant ability was maintained even after prolonged VHT at 150 °C for 16 h or 200 °C for 4 h. Although we could not reveal whether EGCG is converted into GCG after VHT at 150 °C, given the results of antioxidant activity and UV–vis analyses, VHT (at least up to 150 °C) might not cause degalloylation owing to the absence of oxygen, preserving antioxidant activity.

Given the context of our study, the identification of compounds produced from modified EGCG in gelatin after VHT can prove the ability of VHT to prevent the alteration of EGCG. However, this identification faces technical challenges, such as the difficulty in isolating modified EGCG in the pure state. Moreover, heating is expected to promote the denaturation of both base materials (gelatin in our experiments) and modified EGCG, which complicates result interpretation. Heating is known to change the antioxidant activity of gelatin [55]. VHT induces dehydrative condensation and promotes the formation of crosslinked structures in various polymers, such as gelatin [20,56] and collagen [57,58]. Crosslinking modifies the polymers used in biomaterials, altering their stability, solubility, and controlled-drug-release ability [58,59]. Given that heating is expected to affect gelatin properties, we determined the antioxidant activities of sponges with or without EGCG subjected to the same VHT at 150 °C, under conditions that did not affect free EGCG. The radical scavenging ability of EGCG was reported to decrease upon heating under aqueous conditions [13], whereas in our work, modified EGCG retained its antioxidant activity even after VHT at 150 °C for 16 h.

Generally, unheated EGCG has been reported to directly scavenge ROS [38,52] and suppress ROS-producing cells [5]. Previous studies showed that implanted β-TCP is phagocytosed by macrophage-like cells, which then generate ROS [44,60]. Macrophage ROS production is suppressed by unheated free EGCG in vivo [61] and in vitro [62]. The results of our in vitro evaluation indicate that modified EGCG (conjugated to gelatin) maintains its direct ROS-scavenging effect after VHT (Figure 8E,F). Histological examination revealed significantly reduced staining for 4-HNE in β-TCP granules, although the question of whether modified EGCG after VHT removed ROS directly or indirectly via inhibiting ROS-producing cells remains unanswered (Figure 8G,H). However, our results suggest that adequate conditions of VHT preserve the antioxidant ability of modified EGCG.

Our study demonstrates that free and modified EGCG exhibit excellent antioxidant activity after VHT at ≤150 °C. Further research should evaluate physicochemical property changes under prolonged heating and identify the products of decomposition upon VHT at 220 °C and AHT at 200 °C. Moreover, although we used free and modified EGCG (in gelatin), other base materials may have different effects on EGCG stability. Thus, careful consideration is necessary to elucidate the mechanisms of VHT-induced changes in EGCG performance.

## 5. Conclusions

This study evaluated the effects of VHT and AHT on the physicochemical properties and antioxidant activity of free and modified (gelatin-hybridized) EGCG. VHT caused the discoloration and decreased the solubility of free EGCG, with these effects becoming more pronounced with increasing temperature. However, physicochemical and antioxidant property analyses showed no significant changes upon VHT at 150 °C up to 16 h, with marked changes appearing from 220 °C onwards. AHT caused greater changes than VHT, including discoloration and solubility loss, even when performed at 150 °C for 4 h, which indicated that degradation started at lower temperatures under ambient conditions. The AHT-induced decrease in antioxidant activity was partly attributed to decomposition-induced degalloylation. Similar to free EGCG, modified EGCG maintained its antioxidant activity in vivo and in vitro after VHT at 150 °C. Although EGCG is considered a thermally unstable material, our results suggest that free EGCG and EGCG-containing biomaterials subjected to VHT at up to 150 °C may retain their antioxidant capacity and exert pharmacological effects. These insights facilitate the development of novel biomaterials for biomedicine and materials engineering.

## Data Availability

The original contributions presented in the study are included in the article, further inquiries can be directed to the corresponding author.

## Abbreviations

The following abbreviations are used in this manuscript:

EGCG	Epigallocatechin gallate
VHT	Vacuum heating treatment
AHT	Atmospheric heating treatment
DPPH	2,2-Diphenyl-1-picrylhydrazyl
ABTS	2,2′-Azino-bis-3-ethylbenzothiazoline-6-sulfonic acid
UV–vis	Ultraviolet–visible
XRD	X-ray powder diffraction
ATR	Attenuated total reflection
FTIR	Fourier transform infrared
FE-SEM	Field-emission scanning electron microscopy
β-TCP	β-Tricalcium phosphate
NT	Non-heating treatment
EtOH	Ethanol
UPW	Ultrapure water
NMR	Nuclear magnetic resonance
4-HNE	4-Hydroxynonenal
ROI	Region of interest
ANOVA	Analysis of variance
ns	Not significant
GCG	Gallocatechin gallate
EGC	Epigallocatechin
NA	Not available
